# Oncolytic Herpes Simplex Viral Therapy: A Stride toward Selective Targeting of Cancer Cells

**DOI:** 10.3389/fphar.2017.00270

**Published:** 2017-05-16

**Authors:** Dhaval S. Sanchala, Lokesh K. Bhatt, Kedar S. Prabhavalkar

**Affiliations:** Department of Pharmacology, Dr. Bhanuben Nanavati College of Pharmacy, Vile Parle (W)Mumbai, India

**Keywords:** oncolytic viral therapy, herpes simplex virus, cancer, genetic arming, retargeted oncolytic herpes simplex virus-1

## Abstract

Oncolytic viral therapy, which makes use of replication-competent lytic viruses, has emerged as a promising modality to treat malignancies. It has shown meaningful outcomes in both solid tumor and hematologic malignancies. Advancements during the last decade, mainly genetic engineering of oncolytic viruses have resulted in improved specificity and efficacy of oncolytic viruses in cancer therapeutics. Oncolytic viral therapy for treating cancer with herpes simplex virus-1 has been of particular interest owing to its range of benefits like: (a) large genome and power to infiltrate in the tumor, (b) easy access to manipulation with the flexibility to insert multiple transgenes, (c) infecting majority of the malignant cell types with quick replication in the infected cells and (d) as Anti-HSV agent to terminate HSV replication. This review provides an exhaustive list of oncolytic herpes simplex virus-1 along with their genetic alterations. It also encompasses the major developments in oncolytic herpes simplex-1 viral therapy and outlines the limitations and drawbacks of oncolytic herpes simplex viral therapy.

## Introduction

Despite the fact that treatment for cancer has developed phenomenally in the recent years, chemotherapy and radiation therapy remain the pillars of cancer treatment. Chemotherapy and radiation therapy, apart from giving rise to a range of side effects, also have inadequate success for several cancer forms. These shortcomings of the mainstay therapies invite the development of new strategies in the field of cancer. One novel promising approach being the oncolytic viral therapy which uses oncolytic viruses (OVs), a subtype of lytic viruses. OVs destroy tumor cells by selectively replicating in the malignant cells (Mavani and Wick, 2016). Herpes simplex virus (HSV), a neurotropic DNA virus, is one of the most widely studied and used OV. There are two existing variants of HSV viz. HSV type 1 (HSV-1) and HSV type 2 (HSV-2) belonging to family Herpesviridae, subfamily AlphaHerpesviridae. Among the two subtypes, HSV-1 has been studied extensively in cancer oncolytic viral therapy. HSV-1 vectors are classified as replication defective vectors, conditionally replicating vectors and amplicons. Replication defective vectors are consist of transgene expression cassettes, which are introduced into the viral genome having one or more essential viral genes deleted. Such type of HSV-1 vectors efficiently express transgene products, while being incapable to multiply in cells which otherwise do not complement the deleted viral function *in trans*. Conditionally replicating vectors favorably infect, multiply and lyse the malignant cells. Conditionally replicating vectors have deletion of nonessential viral genes. To enhance anticancer activity, conditionally replicating HSV-1 vectors are altered to have therapeutic transgenes (Shen and Nemunaitis, 2006). Amplicons are special vectors which make use of bacterial plasmids to insert transgene cassettes and viral genes essential for signaling which later produces HSV-1 like particles (Federoff et al., 1997; Fraefell et al., 2000; Link et al., 2000). This review focuses on major and recent advances in oncolytic HSV-1 (oHSV-1) viral therapy.

## Advances in oncolytic herpes simplex viral therapy

### Advances in clinical development of oncolytic Hsv

Genetic engineering of OV genome to improve its anti-cancer selectivity has commenced the new era of oncolytic viral therapy. This can be traced to a publication in 1991, wherein thymidine kinase-deleted HSV-1 (dlsptk) attenuated neurovirulence in murine glioblastoma model (Martuza et al., 1991). Two recent Phase 1/2 clinical trials supports efficacy of single agent oncolytic viral therapy, in turn, backed by more than one anecdotal reports (Kelly and Russell, 2007; Park et al., 2008; Senzer et al., 2009; Eager and Nemunaitis, 2011). One of the most convincing evidence of the ability of oncolytic viruses is to efficiently and augment anticancer immunity. Intratumoral injection was provided by a trial which employed a second generation oHSV-1 Talimogene laherparepvec (T-VEC or Imlygic™) formerly called as oncoVEX GM-CSF. T-VEC was administered intratumorally which resulted in total regression of uninjected and injected lesions of 8/50 patients treated with metastatic malignant melanoma (Senzer et al., 2009).

### Increase in Hsv-1 efficacy by increasing its permeability into the tumor cells

The efficiency of OV infection is one of the major hurdle limiting the use of OV therapy for solid tumor malignancy (Shintani et al., 2011). OV therapy employs either direct inoculation or systemic administration of OV. In terms of efficacy, direct inoculation of oHSV-1 is the most reliable technique to dispense oHSV-1 to solid tumors. This is because the combination of leaky vasculature and absence of functional lymphatics in the tumor microenvironment, restricts interstitial transportation and prevents delivery of anti-tumor agents, particularly bulky virus vectors (McKee et al., 2006; Wang and Yuan, 2006). Ultrasound technique, is a therapeutically and diagnostically well established technique for over a decade now (Lindner, 2004; Kaufmann et al., 2007). Ultrasound technique apart from its conventional applications is also used to stimulate cell membrane permeabilization and sonoporation to enhance the anti-cancer efficacy of chemotherapeutic agents and to transfer plasmid DNA *in-vivo* and *in-vitro* setting (Dooley et al., 1983; Fechheimer et al., 1987). A study by Shintani et al. concluded that admission of oHSV-1 into the host Oral Squamous Cell Carcinoma (SCC) Cells was accelerated with the help of ultrasound technique. Furthermore, the presence of microbubbles improved the effect of ultrasound technique (Shintani et al., 2011). These shreds of evidence suggest that ultrasound technique can enhance the efficiency of oHSV-1 infection in OV therapy for oral SCC. Ultrasound technique also has a significant advantage over the rest in being comparatively non-invasive (Lindner, 2004; Kaufmann et al., 2007).

### Systemic delivery of oncolytic Hsv-1 to tumor cells by chelation

Newer strategies are being employed to enhance the systemic delivery of oHSV-1. One recent study on human glioma employing Gli36ΔEGFR, U87ΔEGFR and U251T3 cells demonstrated that chelating copper (Cu) could improve the efficacy by increasing serum stability of oHSV and preventing angiogenesis. The study employed three oHSVs, rHSVQ1, rQnestin34.5, and hrR3, to ensure that effect is not virus strain specific or mutation dependent (Yoo et al., 2012). The increase in Cu levels within the blood serum is noticed in several types of human tumors (Turecký et al., 1984). Cu is a vital co-factor for various angiogenic growth factors, such as angiogenin and Vascular Endothelial Growth Factor (VEGF). In addition to this, Cu is also required by tumor cells to secrete numerous angiogenic factors (Soncin et al., 1997; Hu, 1998). Besides favoring angiogenesis, Cu presence in the blood serum is found to prevent wild-type HSV infection (Shishkov et al., 1997; Panteva et al., 1998; Clewell et al., 2012). Taking into account of the fact that Cu in blood serum prevents wild-type HSV infection, this study was done to investigate whether Cu chelation improves the efficiency of oHSV by increasing its blood serum stability and anti-angiogenic effect. ATN-224, a second-generation copper chelating analog of ammonium tetrathiomolybdate reduced inhibition of oHSV mediated by blood serum. As oHSVs are delivered by intratumoral injection in the clinical setting, these results may provide some impetus for systemic delivery of oHSV as concluded by the study (Yoo et al., 2012).

### Systemic delivery of oncolytic Hsv-1 to the tumor cells by retargeting

**Figure 1:**
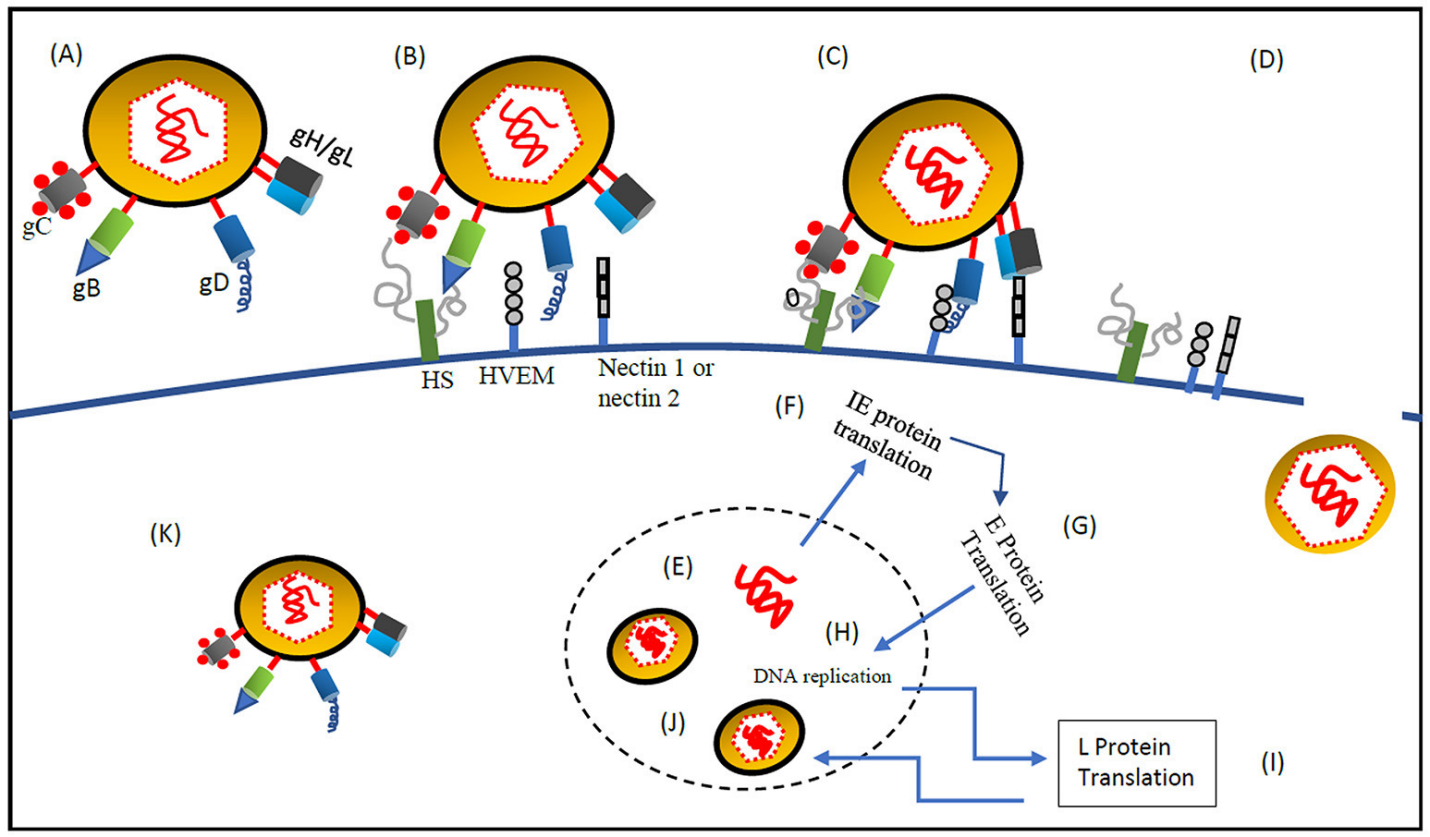
**Systemic delivery of oncolytic HSV-1 to the tumor cells by retargeting. (A)** oHSV-1 contains the following components (1) Core, a dsDNA genome which is opaque to electrons and enfolded as a spool or a toroid. (2) Icosahedral capsid with 162 capsomers surrounding the core. Tegument proteins control the transport of DNA via channels in the capsid (3) Amorphous unstructured matrix of proteins called tegument (4) An outer envelope of lipid bilayer consisting of glycoprotein spikes. The envelope contains 13 types of glycoproteins (Zhou et al., 1999; Shen and Nemunaitis, 2006). **(B)** At first, gC and gB interact with the host cell surface glycosaminoglycan, heparan sulfate (Herold et al., 1991, 1994; Spear et al., 1992). **(C)** Post gC and gB binding, gD interacts with host cell surface receptors, nectin-1a, nectin-1b, nectin-2a, nectin-2d, and HveA, which results in initiation of virions-cell fusion (Spear, 2004). **(D)** Fusion of envelope with host cell membrane and entry of viral DNA and capsid with associated tegument proteins. **(E)** Entry of viral DNA into the host nucleus (Herold et al., 1991, 1994; Spear et al., 1992). **(F)** Once the viral DNA enters the nucleus through the nuclear pore host RNA polymerase II initiates Viral DNA transcription. Five immediate-early (IE) genes, ICP0, ICP4, ICP22, ICP27, and ICP47, are transcribed and translated immediately (Burton et al., 2002) stimulated by VP-16, at the same time host cell shutoff takes place. IE genes are involved in transcription and translation of other HSV genes. **(G)** Before the viral DNA synthesis occurs, Early **(E)** genes transcribe and translate viral SSBP, DNA helicase, origin binding protein, DNA polymerase and localize them into the nucleus. **(H)** DNA replication is origin dependent having three origin sequences. DNA replication takes place in subnuclear constructions called, replication compartments. At the end of the replication cycle, a concatemeric DNA is formed (Mocarski and Roizman, 1982). **(I)** Late gene transcription and translation which produces structural components of the HSV-1 virus. **(J)** Mature virion formation- capsid assembly and encapsidation of concatemeric DNA takes place by systematic cleavage of the concatemeric DNA. Maturation and release of virions is done through Golgi apparatus (egress). **(K)** The fully mature HSV-1 virus is then released from secretory vesicles (Shen and Nemunaitis, 2006).

An important drawback of systemic OV delivery is off target viral replication in patients who are immune compromised. To tackle off target viral replication, one effective approach adopted is retargeting the virus infectivity to attain infection of target cell selectively, see Table 1. Retargeting is done by either, i) Modifying present viral coat proteins or glycoproteins to include single chain antibodies (scFv) or peptide ligands that attach to the required receptor. ii) Utilizing soluble adapters which identify both the oncolytic virus and an exclusive receptor on the target cell. iii) Inserting glycoproteins having distinct host range from other viruses. For retargeting of HSV, several stratagems are employed considering the challenges involved in retargeting HSV. Figure 1 shows process of systemic delivery of oncolytic HSV-1 to the tumor cells by retargeting, which includes eliminating the ingress using the native gD receptors of HSV at the same time attaining infections similar to the ones attained with wt-HSV on vulnerable cells. (Goins et al., 2016). A recent study demonstrated that nectin-1:scFv anti-CEA adapter (soluble bridging molecule) enhances transduction efficacy and produces three times reduction in tumor volume when compared to no adaptor control animals bearing human gastric carcinoma MKN45 tumors in the flank. Infection of HSV-resistant Chinese hamster ovary (CHO) cells expressing ectopic carcinoembryonic antigen (CEA) and vector alone resistant nectin-1/CEA-harboring human gastric carcinoma cells was successfully demonstrated by this adapter (Baek et al., 2011). Another interesting study which employed vascular stomatitis virus-G glycoprotein (VSV-G) to replace either HSV-1 gB (Tang et al., 2001) or gD (Anderson et al., 2000) displayed promising results. When VSV-G was replaced with gD, the entry proficiency of the resultant pseudotyped virus relegated, besides it entered the cell exclusively through endocytosis and most of the virions were trapped and destroyed through the acidic pH of the endosomal pathway. In contrast, when VSV-G was replaced with HSV-1 gB, entry of HSV-1 was comparable with that of wt-HSV in rat striatum. Here VSV-G acts like a fusogen for HSV-1 entrance in the cell due to its structural correspondence to HSV-1 gB. This report gives strong evidence that the HSV-1 entry machinery can be engineered by substituting different glycoproteins with functional substitutes. These in turn produce infectious virions that bring about the cell entry guided by alternate proteins in the cells expressing complementary receptors (Goins et al., 2016).

Table 1**List of oncolytic herpes simplex virus-1 develpoed**.

**Table:** 

**Sr no**.	**Modified HSV-1**	**Genetic modification**	**Transgene inserted**	**Developmental status**
**SINGLE GENE DELETIONS**				
1	dlsptk	*TK^−^*	None	Preclinical
2	hrR3	Disruption of large subunit of ribonucleotide reductase and *UL39*	None	Preclinical
3	HSV1716	*ICP34.5^−/−^*	None	clinical trials
4	R3616	*ICP34.5^−/−^*	None	Preclinical
5	R4009	Premature stop codon in both copies of ICP34.5	None	Preclinical
**MULTIPLE GENES DELETED OHSV-1**				
6	G207	(a) *ICP34.5^−/−^*	None	clinical trials
		(b) Disruption of *UL39*		
7	R7020 (NV1020)	(a) *ICP34.5^−^*	None	clinical trials
		(b) TK under control of viral *a4* promoter		
		(c) *UL24^−^,UL55^−^,UL56^−^*		
8	MGH-1	(a) *ICP34.5^−/−^*	None	Preclinical
		(b) Disruption of UL39		
9	G47Δ	(a) *ICP34.5^−/−^*	None	Preclinical
		(b) Disruption of *UL39* gene		
		(c) *ICP47^−^*		
10	Myb34.5	(a) *ICP34.5^−/−^*	None	Preclinical
		(b) Disruption of UL39 gene		
		(c) Insertion of an *ICP34.5* gene under control of the B-myc promoter		
11	ΔF3y34.5	(a) *ICP34.5^−/−^*	None	Preclinical
		(b) Insertion of an *ICP34.5* gene under control of the DF3/MUC1 Promoter		
**ARMED OHSV-1**				
12	OncoVexGMCSF	(a) *ICP34.5^−/−^*	GMCSF	Approved^#^
		(b) *ICP47^−^*		
13	IL-4 HSV	*ICP34.5^−/−^*	*IL-4*	Preclinical
14	IL-10 HSV	*ICP34.5^−/−^*	*IL10*	Preclinical
15	T-mfIL12	(a) *ICP34.5^−^*	*IL-12*	Preclinical
		(b) *α47^−^*		
		(c) *ICP 6* gene inactivation; HSV-1 strain- HSV G47Δ		
16	NV1042	; HSV-1 strain- NV1023	*IL-12*	Preclinical
17	bG47Δ-PF4	(a) *ICP34.5^−/−^*	*PF4*	Preclinical
		(b) Disruption of *UL39* gene		
		(c) *ICP47^−^*.; HSV-1 strain- HSV G47Δ		
18	T-TSP-1	(a) *ICP34.5^−/−^*	*TSP-1*	Preclinical
		(b) *ICP6^−^*		
		(c) *α47^−^*		
19	RAMBO	(a) *ICP34.5^−/−^*	*Vstat120*	Preclinical
		(b) Possesses a gene disrupting insertion of green fluorescent protein within the viral UL39 locus encoding for ICP6 gene; HSV-1 strain- HSVQ		
20	OSVP	(a) *OS^−^*	*15-PGDH*	Preclinical
		(b) vhs; *UL41^−^*; HSV-1 strain- OSV		
21	HSV1Ycd	(a) *ICP6*^−^; HSV-1 strain- bMP6-CMVFcumB	*yCD*	Preclinical
22	1- vHsv-B7.1-Ig 2- vHsv-IL-12 3- vHsv-IL-18	(a) *ICP34.5^−/−^*		Preclinical
		(b) Insertion of Green fluorescent protein gene in ICP locus; HSV-1 strain-vHsv-IL-18	(a) *B7.1-Ig* (b)*IL-12* (c)*IL-18*	
23	G47Δ-IL18/B7	(a) *ICP34.5^−^*	*IL-18* and soluble *B7-1*	Preclinical
		(b) *α47^−^*		
		(c) *ICP6*^−^; HSV-1 strain-G47Δ		
24	rQT3 and rQLuc	(a) *ICP34.5^−^*	*TIMP3* or firefly *luciferase*	Preclinical
		(b) *ICP6*^−^; HSV-1 strain- rHSVQ1		
	−	(a) *ICP34.5^−^*	*GALV.fus or EGFP*	Preclinical
		(b) fHSV-delta-pac^−^

**Table:** 

**RETARGETED ONCOLYTIC HSV**							
		**gB**	**gC**	**gD**	**gH/gL**		
**A. Fusogenic membrane glycoproteins (FMGs)**							
25	DUs3-8	−	−	HveA^−^/HveC^−^ ^*^	−	VSV-G	Preclinical
26	Amplicon VSV-G	Δ gB	−	−	−	VSV-G	Preclinical
**B. Peptide ligands**							
27	KgBpK:-gC-EPO	HS^−^	HS^−^ ^*^	−	−	full-length erythropoietin hormone EPO	Preclinical
28	KgBpK-:gC-preS1	HS^−^	HS^−^ ^*^	−	−	HBV-sAg	Preclinical
29	R5111	HS^−^	HS^−^ ^*^	HveA^+^/HveC^+^ ^*^	−	IL-13	Preclinical
30	R5141	HS^−^	HS^−^ ^*^	HveA^−^/HveC^−^ ^*^	−	IL-13	Preclinical
31	R5181	HS^−^	HS^−^ ^*^^1^	HveA^+^/HveC^+^ ^*^^2^	−	IL-13^*^^1^and uPA^*^^2^	Preclinical
32	AmpliconpCONGA-H	−	HS^−^ ^*^	−	−	HIS-tag	Preclinical
33	Amplicon pCONGA-MG11	−	HS^−^ ^*^	−	−	MG11 peptide	Preclinical
34	AmplicongC-BDNF	HS^−^	HS^−^ ^*^	−	−	pre-pro BDNF	Preclinical
35	AmplicongC-GDNF	HS^−^	HS^−^ ^*^	−	−	pre-pro GDNF	Preclinical
36	Amplicon NMDA NR2A/2B	HS^−^	HS^−^ ^*^	−	−	NMDA NR2A/B Ab	Preclinical
**C. Single chain antibodies (scFvs)**							
37	KGNEp	NA	−	HveA^−^/HveC^−^ ^*^	−	scFvEpCAM	Preclinical
38	KGNE	NA	−	HveA^−^/HveC^−^ ^*^	−	scFv EGFR	Preclinical
39	KGNC	−	−	HveA^−^/HveC^−^ ^*^	−	scFv CEA	Preclinical
40	R-LM113	−	−	HveA^−^/HveC^+^ ^*^	−	scFv HER2	Preclinical
41	R-LM249	−	−	HveA^−^/HveC^−^ ^*^	−	scFv HER2	Preclinical
42	R-VG809	−	−	HveA^−^/HveC^−^ ^*^	−^*^	scFv HER2	Preclinical
43	R-LM31	−	−	−^*^	−	scFv HER2	Preclinical
44	R-LM11	−	−	HveA^−^/HveC^+^ ^*^	−	scFv HER2	Preclinical
45	R-LM39	−	−	HveA^−^/HveC^+^ ^*^	−	scFv HER2	Preclinical
46	HSV1716 scFv CD55	−	−	HveA^−^/HveC^−^ ^*^	−	scFv CD55	Preclinical
47	Amplicon pCONGA-MR1-1	HS^−^	HS^−^ ^*^	−	−	scFv EGFR MR1-1	Preclinical
**D. Adapters**							
48	HVEM: CEA Adapter	−	−	−	−	scFv CEA	Preclinical
49	Nectin1: EGFR Adapter	−	−	−	−	scFv EGFR	Preclinical
50	Nectin1 Adapter	−	−	−	−	nectin1-HveC	Preclinical

* Transgene molecule inserted

# Imlygic™

−*Wild type*

*RAMBO, Rapid Antiangiogenesis Mediated By Oncolytic virus; HVEM:CEA, Herpes Virus Entry Mediator; Carcino Embryonic Antigen; TK, Thymidine Kinase; OS, OncSyn; HS, Heparan Sulfate; HveA, Herpes Virus Entry Mediator A; HveC, Herpes Virus Entry Mediator C; gB, glycoprotein B; gC, glycoprotein C; gD, glycoprotein D; gH/gL, glycoprotein H/L; IL4, Interleukin 4; IL10, Interleukin 10; IL12-Interleukin 12; IL13, Interleukin 13; GM-CSF, Granulocyte Macrophage Colony Stimulating Factor; IL18, Interleukin 18; PF4, Platelet Factor 4; TSP-1, Thrombospondin-1; Vstat, Vasculostatin; 15-PGDH, 15-Prostaglandin Dehydrogenase; yCD, yeast Cytosine Deaminase; GALV.fus, gibbon ape leukemia virus envelope fusogenic glycoprotein; EGFP, enhanced green fluorescent protein gene; VSV-G, vesicular stomatitis virus glycoprotein G; EPO, Erythropoietin; EGFR, Epidermal Growth Factor Receptor; ICP, Infected Cell Protein*.

### Systemic delivery of retargeted oncolytic Hsv to the tumor cells by employing carrier

Retargeted oHSVs have been proven efficacious when it comes to systemic delivery of oHSV. To further increase the effectiveness of retargeted oHSVs, the viral protein cargo is packaged with the help of carrier cells to deliver it effectively to the tumor site. The early research employed irradiated tumor cell, which was later discontinued taking in safety considerations (Munguia et al., 2008; Park et al., 2008; Russell and Peng, 2008; Willmon et al., 2009). The packaging of oHSVs with the carrier can be done either by loading onto the cells or by incorporating into the cell's compartments. The virus particles can be delivered by the carrier cells in three ways, (a) Tumor microenvironment can be used to attract carrier cells. (b) Cytokine-induced killer carrier cells or tumor specific carrier T cells can be used to target tumor cells particularly. (c) In the third method the tumor cells are not targeted by the carrier cell, but the tumor location is targeted e.g., lymph nodes. Mesenchymal stem cells appear to be promising carriers belonging to the former group wherein they are accumulated in the tumor stroma due to the tumor microenvironment having the hypoxic environment and inflammatory cytokine expression associated with the tumor. Limitations being the prevention of virus infection to the carrier cells due to the oHSV-1 specificity and mesenchymal stem cell transferred adoptively may itself feed the tumor (Willmon et al., 2009). A study in 2015 showed delivery of R-LM249 via MSCs is achievable and efficient. One problem faced was MSCs could be barely infected with HER-2 retargeted R-LM249, but the problem was solved by forcibly infecting MSCs with retargeted R-LM249. Also, therapeutic results were reaped with single i.v dose of R-LM249 infected MSCs in lung and brain metastases. The study concluded achievable and efficient delivery of carrier-mediated (MSCs) delivery of retargeted oHSV (Leoni et al., 2015).

### Systemic delivery of oncolytic Hsv to the tumor cells by minimizing sequestration in the liver and spleen

Pre-treating blood serum with cyclophosphamide/venom of cobra to exhaust blood serum factors like complement proteins and IgM to which HSV binds readily decreases sequestration and increases systemic availability (Chiocca et al., 1999; Ikeda et al., 2000; Wakimoto et al., 2002). A recombinant HSV-1, hrR3, with U_L39_ deleted, selectively replicates in cancerous cells with significant anticancer activity and results in better survival in animal models of brain (Mineta et al., 1994; Jacobs et al., 2001), pancreas (Kasuya et al., 1999), colon (Yoon et al., 2000) and liver cancers (Pawlik et al., 2000). It was conjectured that a strong immune suppressant like cyclophosphamide might ad interim repress systemic immunity for viruses, permitting better replication of the therapeutic virus and augment the anticancer effect (Ikeda et al., 1999). Co-administration of cyclophosphamide, an antineoplastic and immune suppressive agent, proved to be efficacious as it improved the anticancer activity of hrR3 (Ikeda et al., 1999; Wakimoto et al., 2004).

### Intratumoral oncolytic virus spread

In the case of solid tumor malignancies, it is not considered advantageous to dispense OV by systemic route considering that the tumor is usually isolated from general systemic circulation and delivering the OV systemically subjects patient's full body to the virus inflicting the entire body to the danger of undesirable clinical circumstances. As cancer patients already have suppressed systemic immunity, administration of virus might trigger clinical complications which might not appear in healthy animals (Coffey and Thompson, 2002). Intratumoral injection of oHSV-1 is one of the most reliable techniques of administering oHSV-1 (Shintani et al., 2011). It was believed that injecting OV intratumorally results in the generation of more virus particles by the malignant cells and the newly generated virus particles shortly replicate in every individual tumor cell by expanding through the tumor. Soon it was found that reovirus killed malignant cells and brought about local necrosis on single intratumoral injection while the tumor cells distant from the injection site continued to proliferate indicating the spread of the virus was not efficient enough. These finding sparked the need to developed new strategies of intratumoral injection to increase virus spread. One such strategy was drawn up to deliver HSV-1 vectors. The strategy consisted of OV delivery to multiple tumor sites or the OV could be injected intratumorally at a single site but in a volume large enough which is capable of getting to more malignant cells. When the OV is being injected at multiple sites inside the tumor mass, it is advised that at least 3–5 sites are injected. In the case of single injection at a single site, the composition of OV should be between 10 and 100% of the tumor mass volume (Coffey and Thompson, 2002). The tumor microenvironment is dense in connective tissue (Sauthoff et al., 2003; Yun, 2008). Losartan, U.S FDA approved Ag-II antagonist improves the reach of oHSV into the tumor mass via disturbing transforming growth factor beta 1 (TGF-β1) signaling and subsequently declining production of stromal collagen in the tumor microenvironment of human breast, pancreatic and skin desmoplastic tumor models in mice (Diop-Frimpong et al., 2011).

### Chemical sensitizers to enhance oncolytic virus growth in tumor cells

A neoteric approach to enhance oHSV-1 development in malignant cells is by using chemical sensitizers. Small molecules as chemical sensitizers neutralize the residual antiviral activities within the tumor cells that are resistant to the OV. Histone deacetylase (HDAC) inhibitors were shown to repress residual Interferon (IFN) responsiveness by the tumor cell in turn enhancing the efficacy of oHSV-1 with no compromise in the oHSV-1 specificity (Chang et al., 2004; Nguyên et al., 2008; Otsuki et al., 2008; MacTavish et al., 2010). A couple of small molecules, dilazep and dipyridamole, were identified in a high throughput screening with attenuated HSV lacking ribonucleotide reductase. Both the small molecules improved virus growth and decreased tumor growth in athymic mice bearing subcutaneous Du145 tumors by inhibiting cellular equilibrative nucleoside transporter-1 (ENT-1) eventually resulting in induction of cellular ribonucleotide reductase (Passer et al., 2010).

### Enhancing anti-tumor immunity

A key hallmark of cancer, immune escape by the tumors, presents a valuable target in the field novel cancer therapeutics. HSV-1 armed with GM-CSF (T-VEC) proved to be successful in treating cancers all due to the recruitment of immune cells coupled with confined oncolytic activity (Burke, 2010; Melcher et al., 2011). Phase I clinical trial of HSV armed with GM-CSF was conducted in patients with either cutaneous or s.c gastrointestinal, breast and head and neck deposits of cancer resulted in extensive immune cell infiltration as revealed by post-treatment biopsies (Hu et al., 2006). A range of cytokines have been used with oHSV to modify and enhance antitumor immunity including GM-CSF, IL12, IFN-α and Tumor necrosis factor (TNF-α) but GM-CSF has reproduced the most reliable and best results. IL-12, IFN-α and TNF-α have also shown promise in preclinical cancer studies (Liu et al., 2003). A study in murine HER-2/neu breast cancer cells, TUBO, *in-vitro* and *in-vivo* employed HSV-1 and HSV-2 *ICP0* mutants to investigate the effect of oncolytic viruses on the immune system. The concluded, extended replication of the oncolytic virus within the tumor cells is insignificant, but the initial stages of immunogenic virus replication are more important which leads to activation of antitumor immunity (Workenhe et al., 2014). A study on A20 lymphoma tumors in BALB/c mice reported that oncolytic HSV-1 (GM-CSF inserted into ICP34.5/ICP47 deleted strain of HSV-1, JS1) serves as patient-specific tumor vaccine by the liberation of tumor antigens when administered intratumorally. The virus had antitumor immune effects on both injected and uninjected tumors (Liu et al., 2003). A recent interesting study reported that oHSV-2 can induce cell death of colorectal cancer cells both *in-vitro* and *in-vivo* along with recruiting adaptive immune response (Yin et al., 2017).

### Genetic arming

HSVs are “armed” with genes that can enhance antitumor cytolytic activity. Most commonly oHSV-1 is armed with immunostimulatory molecules. Apart from immunostimulatory molecules oHSV-1 is also armed with prodrug converting enzymes, angiogenesis inhibitors, suicide genes and fusogen membrane glycoproteins (Shen and Nemunaitis, 2006), see Table 1. (i) Oncolytic HSV-1 armed with Immunomodulatory molecules- oHSV-1 armed with soluble *B7-1, IL-12* or *IL-18* were developed and displayed similar replication profile as to the wt-HSV *in-vitro*. The *in-vivo* efficacy was evaluated in A/J mice bearing syngeneic, poorly immunogenic tumors of Neuro2a neuroblastoma. *IL-12* happened to have the greatest efficacy among the three expressed by G207 (double mutated oHSV-1). The triple combination proved to be more efficient than either double or single combination of the armed oncolytic viruses (Ino et al., 2006). (ii) Oncolytic HSV-1 armed with anti-angiogenic molecule- rQt3, an oHSV-1 armed with metalloproteinases 3 tissue inhibitor. rQt3 produced immature collagen extracellular matrix increasing peak infection level of the virus with a remarkable reduction in tumor vascular density resulting in decreased endothelial progenitor cell circulation and delayed the growth of the tumor in athymic mice carrying malignant peripheral nerve sheath or human neuroblastoma tumor (Mahller et al., 2008). (iii) Oncolytic HSV-1 armed with fusogenic membrane glycoprotein: arming oHSV-1 with fusogenic membrane glycoproteins might result in syncytium formation by the neighboring uninfected cells, in turn, facilitating the killing of the tumor cell. There are some toxicity concerns on the uninfected tissues (Todo, 2008). (iv) Oncolytic HSV-1 armed with Suicide genes/Pro-drug converting enzymes: an oHSV-1 (MGH2) genetically modified to express two prodrug-activating genes, secreted human intestinal carboxylesterase and CYP2B, wherein each yields active metabolite of irinotecan and cyclophosphamide. The study concluded, MGH2 proves to be effective in preclinical glioma models in combination with chemotherapeutic agents (Tyminski et al., 2005). (v) Oncolytic HSV armed for *in-vivo* non-invasive imaging. Two oHSV-1 were created expressing *firefly luciferase* with either immediate early 4/5 promoter or *gC* promoter for non-invasive *in-vivo* imaging. Athymic mice carrying subcutaneous Gli36ΔEGFR glioma tumors were then studied with super sensitive charged coupled device camera for luciferase expression. Luciferase expressed by *IE4/5* promoter was linked with oHSV-1 infection as *IE4/5* promoter acts immediately post viral infection. Luciferase expressed by strict late *gC* promoter is associated with viral replication as strict late *g/C* promotor, late in the replication cycle (Yamamoto et al., 2006).

## Conclusion

HSV-1 is one of the foremost OV in oncolytic therapeutics. Advances in oHSV-1 therapy during the last decade indicate its promising future. The oHSV-1 is considered to be relatively safe as it does not integrate into the host genome unlike adenovirus and thus eliminates the risk of any insertional mutagenesis in the host genome. The oHSV-1 has proved to be a valuable therapeutic option for controlling cancers of not only CNS origin but also other tissue of the body, such as skin and colon. In the year 2015, the first oHSV-1 was approved (Talimogene laherparepvec or T-VEC) for melanoma by the trade name Imlygic™ and shows promise till date. This indicates that oHSV-1 viral therapy is efficient and is a novel anticancer therapy. Several other non-HSV oncolytic viruses are making headway toward approval. Vaccinia virus with genetically modified thymidine kinase and human GM-CSF, LacZ insertion (Jx-594/Pexa-vec/pexastimogene/devacirepvec) is being evaluated in Phase III clinical trial for Advanced-stage hepatocellular carcinoma. Adenovirus with genetically modified E2F1 promoter/E1A gene and human GM-CSF insertion (CG0070) is being evaluated for non-muscle invasive bladder cancer after BCG failure in Phase II/III clinical trial. Reovirus as reolysin (pelareorep) has completed Phase III clinical trial with orphan drug designation from US FDA for the treatment of metastatic and/or recurrent head and neck cancer. Moreover, additional in-depth insight to the virus biology will help to add more useful modifications that can prove advantageous in cancer therapeutics.

## Author contributions

DS: contributed toward data collection and writing the paper. LB: contributed toward conceptualization, planning and writing the paper. KP: contributed data collection and writing the paper.

### Conflict of interest statement

The authors declare that the research was conducted in the absence of any commercial or financial relationships that could be construed as a potential conflict of interest.
